# Hsa_circ_0001859 promotes NSCLC progression through the miRNA-101-3p/MMP1 axis

**DOI:** 10.3389/fonc.2025.1568367

**Published:** 2025-07-01

**Authors:** Jianxin Tan, Zhenyu Fan, Rongguo Lu, Shugao Ye

**Affiliations:** ^1^ Lung Transplant Center, Department of Thoracic Surgery, The Affiliated Wuxi People’s Hospital of Nanjing Medical University, Wuxi People’s Hospital, Wuxi Medical Center, Nanjing Medical University, Wuxi, Jiangsu, China; ^2^ Department of Infectious Disease, The Affiliated Wuxi People’s Hospital of Nanjing Medical University, Wuxi People’s Hospital, Wuxi Medical Center, Nanjing Medical University, Wuxi, Jiangsu, China

**Keywords:** non-small cell lung cancer, circ_0001859, miR-101-3p, MMP1, progression

## Abstract

**Background:**

Non-small cell lung cancer (NSCLC) is a prevalent form of lung cancer characterized by a significant incidence and mortality rate in China. Most patients are diagnosed at an advanced stage. Circ_0001859 served as an exon circular RNA, but its specific role in NSCLC remained extensively unexplored.

**Methods:**

Quantitative Real-Time-Polymerase Chain Reaction (qRT-PCR) and western blotting were utilized to detect messenger RNA (mRNA) and protein expression levels in NSCLC tissues and cells. Cell proliferation was assessed by Cell Counting Kit-8 (CCK-8) and colony formation assays. Cell migration and invasion were evaluated by transwell assay. Mechanistically, the mechanisms and target binding relationship between circ_0001859, miR-101-3p and MMP1 were assessed through the luciferase reporter and RNA immunoprecipitation (RIP) assays. *In vivo* xenograft model was established to examine the impact of circ_0001859.

**Results:**

Circ_0001859 was significantly overexpressed in NSCLC tissues and cells. Functional studies demonstrated that silencing circ_0001859 significantly impeded the malignant phenotype of NSCLC cells. Bioinformatics analysis and rescue experiments disclosed that circ_0001859 functioned as a sponge for miR-101-3p, modulating *MMP1* expression and thereby controlling NSCLC development and metastasis. The role of circ_0001859/miR-101-3p/MMP1 axis was validated in xenograft tumor models *in vivo*.

**Conclusions:**

Our research findings demonstrated that circ_0001859 engaged in regulating NSCLC growth and metastasis via the miR-101-3p/MMP1 pathway. These studies presented the first investigation into the precise function and putative regulatory mechanism of circ_0001859 in NSCLC, providing valuable insights towards prognostic indicators and therapeutic targets for malignant tumors.

## Introduction

Lung Cancer (LC), one of the most prevalent malignant tumors worldwide, accounts for more than 2.4 million new cases annually, representing 12.4% of the total cancer cases, and causing the deaths of nearly 1.8 million patients (1). Among them, non-small cell lung cancer (NSCLC) accounts for the 80-85% of lung cancer cases (2), with lung adenocarcinoma (LUAD) and lung squamous cell carcinoma (LUSC) being the predominant subtypes (3). Recently, substantial advancements have been achieved in diagnosing and treating NSCLC owing to the widespread implementation of early tumor screening and precision medicine (4, 5) Nevertheless, owing to factors like subtle symptoms, insufficient early diagnostic tools, delayed detection, etc., approximately 75% of patients are diagnosed in advanced stages of the disease, frequently presenting with distant metastases, hence losing the prime window for surgical intervention (6). Patients suffering from lung cancer generally exhibit a dismal prognosis post-treatment, as evidenced by a five-year survival rate that stands at roughly 23% (7).

Circular RNAs (circRNAs) are novel non-coding RNAs characterized by a circular structure that are widely present in eukaryotic cells and originate from the backsplicing of precursor messenger RNAs (mRNAs) (8). Accumulating evidence demonstrates that circRNAs play regulatory roles in various cellular processes, and their dysregulation has been associated with multiple diseases, including cancers (9), immune disorders (10) and cardiovascular diseases (11). In oncology, circRNAs have been shown to regulate cell proliferation, migration, angiogenesis, and stemness in various tumor types, and the underlying mechanisms are being extensively investigated. The underlying mechanisms are being extensively investigated. These findings provide directions for novel strategies for tumor treatment and innovative tumor biomarkers. For instance, circ_PPAPDC1A functioned as a sponge for miR-30a-3p to active IGF1R/PI3K/AKT/mTOR pathway, exerting an oncogenic role in NSCLC with Osimertinib resistance (12). Notably, circUSP7 was enriched in exosomes secreted by NSCLC and regulated the downstream *SHP2* expression by sequestering miR-934, ultimately leading to functional dysregulation of CD8+ T cells, immune evasion, as well as resistance to anti-PD-1 therapy in NSCLC (13). Circ_0000190 directly involved in EGFR-MAPK-ERK signaling to promote NSCLC progression, emphasizing the potential of inhibiting circ_0000190 as a therapeutic strategy against NSCLC (14). Circ_0001859, an exon-derived circRNA, has been primarily studied in neuronal injury (15) and cardiac hypertrophy (16), while its function in cancer remains limited. Li at al (17). investigated that circ_0001859 regulated ATF2 expression by sponging miR-204/211, thereby playing an important role in the pathogenesis of chronic inflammatory diseases in synovial tissue. Nonetheless, comprehensive understanding of circRNA_001859 expression and specific function in NSCLC remain largely unexplored. Therefore, there exists an imperative necessity for in-depth exploration in this domain.

MicroRNAs (miRNAs) are small (10–22 nucleotides), evolutionarily conserved, non-coding RNAs that post-transcriptionally regulate approximately one-third of human genes through mRNA degradation or translational suppression. Since their discovery, miRNAs have been established as key regulators that function as either oncogenes or tumor suppressors in cancer development by binding to the 3’-UTR of target mRNAs. Increasing evidence suggested the reciprocal interactions between circRNAs and mRNAs, forming a complex network of gene-targeted regulation. For instance, circ_0001671 acted as a competing endogenous RNA by sponging miR-27b-3p, thereby reducing BLM expression and inhibiting disease progression in gastric cancer (18). Therefore, further functional studies are required to enhance our comprehension of their mechanistic functions.

The matrix metalloproteinase (MMP) family comprises a group of metalloproteinases that degrade proteins in the extracellular matrix (19). As the prototypical member, MMP1 has been extensively implicated in tumor cell invasion and metastasis due to its dysregulated expression (20). MMP family proteins exhibit an abnormal expression pattern in a variety of malignant solid tumor tissues, including breast cancer (21), NSCLC (22), cervical cancer (23), and colorectal cancer (24). For instance, in head and neck squamous cell carcinoma (HNSCC), MMP9 and MMP2 exhibited a synergistic interaction that critically regulates tumor progression through coordinated modulation of cellular proliferation, migration, and invasive processes (25). CTHRC1 functioned as a pro-metastatic driver in NSCLC, orchestrating tumor invasion and metastatic dissemination through transcriptional upregulation of matrix metalloproteinases MMP7 and MMP9 (26). Hence, further comprehensive exploration is warranted.

In this study, we observed circ_0001859 was significantly upregulated in NSCLC tissues and cells. Silencing its expression alleviated the malignant phenotype in NSCLC cells. Mechanistically, we established circ_0001859 as a molecular sponge for miR-101-3p, thereby regulating MMP1 expression. These findings provide novel molecular targets for NSCLC diagnosis, prognosis assessment, and targeted therapy development.

## Materials and methods

### The patient sample

Cancer tissue samples were collected from NSCLC patients who underwent surgical treatment at our institute, along with corresponding adjacent non-cancerous tissue. All patients were diagnosed with NSCLC based on preoperative pathology and had not received chemotherapy or radiation therapy. Pathological classification and tumor staging were performed according to the Union for International Cancer Control criteria. Of the 40 patients, 16 had stage I–II disease and 24 had stage III–IV disease, with 28 patients exhibiting lymph node metastasis (LNM). All tissues were stored in ultra-low temperature freezer for subsequent examination. All patients signed informed consent forms and this study was approved by the Ethics Committee of The Affiliated Wuxi People’s Hospital of Nanjing Medical University.

### Cell culture and transfection

The human LUAD cell line CALU3, human NSCLC cell lines A549 and H1299, human large cell lung cancer cell line H460, and the normal human bronchial epithelial cell line 16HBE were purchased from the Cell Bank of Chinese Academy of Sciences (Shanghai, China). The four cell lines are considered representative of NSCLC owing to their widespread applicability and accessibility. All cell lines were incubated in an RPMI-1640 (Gibco, Grand Island, CA, USA) medium supplemented with 10% fetal bovine serum (FBS) (Thermo Fisher Scientific, Waltham, MA, USA) and 1% penicillin-streptomycin antibiotics (Gibco). The cells were cultured at 37°C in a CO_2_ incubator. NSCLC cells were cultured to the logarithmic growth phase prior to transfection. sh-circ_0001859#1, sh-circ_0001859#1, and their control (sh-NC), as well as the miR-101-3p inhibitor/mimic and the mimic negative control were synthesized by Ribobio Inc. (Guangzhou, Guangdong, China). Transfection was performed using Lipofectamine 3000 reagent (Invitrogen, Carlsbad, CA, USA) following the manufacturer’s instructions for 48 hours. The transfected cells were collected for further research. The relevant sequences are mentioned in **Supplementary Table S1**.

### Quantitative Real-Time-Polymerase Chain Reaction (qRT-PCR)

Total RNAs were extracted from NSCLC tissues and cells using TRIzol kit (Invitrogen) according to manufacturer’s instructions. Concentration of RNA was determined using Nanodrop 2000 (Thermo Fisher Scientific). Subsequently, PrimeScript™ RT reagent Kit (Takara, Otsu, Shiga, Japan) was utilized to reverse transcribe 1000 ng of total RNA from tissues or cells for subsequent cDNA synthesis. Subsequently, qRT-PCR analysis was performed to assess the expression levels of circ_0001859, *MMP1*, and miR-101-3p. Specifically, the reactions utilized the SYBR Green PCR Master Mix (Thermo Fisher Scientific), with cDNA as the template for PCR amplification. All assays were conducted on Real-Time PCR Systems (Applied Biosystems, Foster City, CA, USA). The thermocycling conditions were as followed: 95°C for 15 min, followed by 40 cycles at 94°C for 15 s, 55°C for 30 s, and 70°C for 30 s. GAPDH and U6 were served as internal reference for mRNA and miRNA. Record the Ct values of the target gene and the Ct values relative to the reference gene displayed on the instrument, and calculate the ΔCt value. Then, calculate ΔΔCt using the formula ΔΔCt = ΔCt (experimental group) − ΔCt (control group). Relative expression levels were calculated according to the 2^−ΔΔCt^ method, and perform normalization processing on the data. Primer sequences were listed as followed (5′→3′).

circ_0001859-F: GCCGAGAGAGAGTCCAGTCTT
circ_0001859-R: AAAGGGTCACAGCTCCCGAA
miR-101-3p -F: ACGGGCGAGCTACAGTACTGTG
miR-101-3p -R: CCAGTGCAGGGTCCGAGGTA
*MMP1*-F: GAAGGTGAAGGTCGGAGTC
*MMP1*-R: GAAGATGGTGATGGGATTTC
GAPDH-F: AATCCCATCACCATCTTC
GAPDH-R: AGGCTGTTGTCATACTTC
U6-F: CGCTTCGGCAGCACATATAC
U6-R: CAGGGGCCATGCTAATCTT

### Subcellular RNA fractionation experiment

Nuclear and cytoplasmic RNA were extracted from NSCLC cells using a nuclear/cytoplasmic fractionation kit (Norgen Biotek, Thorold, ON, CAN) according to the manufacturer’s protocol. Subsequently, the isolated nuclear or cytoplasmic RNA was analyzed by qRT-PCR to measure the expression levels of the nuclear internal control (U6), cytoplasmic internal control (GAPDH), and the circular RNA circ_0001859.

### Cell counting kit-8 assay

Cell proliferation was determined using a CCK-8 assay (Beyotime, Beijing, China). Cells were seeded onto a 96-well plate at a density of 1×10^3^ cells/well and incubated for 0, 24, 48, and 72 hours. Subsequently, the cells were rinsed with PBS and then treated with 10 μL CCK-8 solution, followed by incubation at 37°C for 2 hours. The absorbance of the cells at 450 nm was quantified using a microplate reader.

### Colony formation assay

Cell proliferation was assessed by calculating the colony forming efficiency. Transfected and non-transfected cells (300 cells/well) were seeded into separate wells of a 6-well plate and cultured for 12 days at 37°C. Cells were then fixed with 4% paraformaldehyde (Beyotime) and stained with 0.1% crystal violet (Solarbio, Beijing, China) for 30 minutes. Finally, visualize and manually count visible colonies under a microscope (Olympus, Tokyo, Japan).

### Migration and invasion assays

Transwell assays with or without Matrigel-coated membranes was performed to measure cells invasion and migration abilities. Briefly, Matrigel (BD Biosciences, Franklin Lakes, NJ, USA) was diluted with serum-free medium and evenly coated on the upper chambers of 24-well Transwell plates to form a matrix barrier. Serum-free medium containing 200 μL NSCLC cells (1×10^5^ cells/mL) was added to the upper chamber, while 500 μL medium supplemented with 10% FBS (Solarbio) was added to the lower chamber. The plates were then incubated at 37°C for 48 hours, followed by washing with PBS (Solarbio) and fixation with 4% paraformaldehyde (Solarbio). Subsequently, the cells were stained with 0.1% crystal violet for 20 minutes. Microscopy imaging and manually count to measure cells invasion capability. However, for cell migration assessment, the Matrigel coating step was intentionally excluded.

### Western blotting analysis

Proteins from NSCLC cell lines were extracted using RIPA lysis buffer, and the quantification was performed using the Bicinchoninic Acid (BCA) Assay Kit (Beyotime). The proteins were then separated by 12% SDS-PAGE gel and transferred to the PVDF membranes, which was subsequently blocked with 1% BSA solution at room temperature for 2 hours. Primary antibodies against *MMP1* (1:1000, ab134184, Abcam, Cambridge, MA, USA), Snail (1:1000, ab216347, Abcam), N-cadherin (1:1000, ab245117, Abcam), E-cadherin (1:1000, ab76055, Abcam), Vimentin (1:1000, ab92547, Abcam), and GAPDH (1:2500, ab9485, Abcam) were added and incubated overnight at 4°C. Then, the membranes were incubated with a goat anti-rabbit IgG (1:2000, ab6721, Abcam) antibody at room temperature for 2 hours. Finally, the bands were covered with an ECL chemiluminescent reagent (Beyotime) and visualized using a ChemiDoc XRS+S system (Bio-Rad, Hercules, CA, USA). Finally, grayscale intensity was analyzed using Image J software (NIH, Bethesda, MD, USA) to quantify protein expression.

### Dual-luciferase reporter assay

Utilizing the starBase2.0 for prediction, miRNAs targeting circ_0001859 and its downstream mRNAs were predicted. Fragments of circ_0001859 and *MMP1*, either with the wild-type (WT) or mutant (MUT) form, were cloned into the firefly luciferase reporter pGL3 vector (Promega, Madison, WI, USA). Subsequently, the constructed vectors were co-transfected into A549 and H1299 cells with miR-101-3p mimic, miR-101-3p inhibitor, or mimic NC using Lipofectamine 3000 reagents. Cells were collected 48 hours post-transfection, and luciferase activity was measured using the Dual-Luciferase Reporter Assay System (Promega). Each experiment was replicated three times. The sequences of luciferase constructs circ_0001859-WT, circ_0001859-MUT, *MMP1*-WT and *MMP1*- MUT are summarized in **Supplementary Table S1**.

### RNA immunoprecipitation assays

The RIP assay kit (17-701, Millipore, Billerica, MA, USA) was utilized to investigate the interaction between circ_0001859 and miR-101-3p. Cells were lysed using RIP lysis buffer. Subsequently, the lysate was incubated overnight at 4°C with magnetic beads that were pre-coated with either anti-Ago2 (1:50, ab32381, Abcam) antibody or anti-IgG (1:100, ab109489, Abcam) antibody. The magnetic beads were collected through centrifugation. Finally, qRT-PCR was employed to detect miR-101-3p levels enrichment in immunoprecipitated RNA.

### Mice xenograft assay

A total of 12 male nude BALB/c mice (age, 4–5 weeks; weight, 20–25 g) were used in this study. After one week of acclimatization, the mice were randomly divided into two groups, with 6 mice in each group. A549 cells were transfected with 100-nM circ_001859 inhibitor (sh-circ 001859#1) or negative control (sh-NC) for 48 hours. Subsequently, the cells were collected and subcutaneously injected into the dorsal sides of mice at a density of 5 × 10^6^ cells. The length and width of tumors were measured every 5 days, and tumor volume was calculated as follows: (length × width^2^)/2. On day 25, the mice were euthanized and tumors were excised for imaging and subsequent experiments. All experimental procedures involving animals were approved by the Animal Ethics Committee.

### Statistical analysis

The GraphPad Prism (version 8.0) (GraphPad, San Diego, CA, USA) and SPSS Statistics (version 18.0) (SPSS, Chicago, IL, USA) software were used for graphical and statistical analyses, respectively. All data were expressed as the mean ± standard deviation (SD). All experiments were repeated at least 3 times. Significant differences between different experimental groups were analyzed by one-way analysis of variance (ANOVA). Group comparisons were performed using t-test for assessing significance. *P* < 0.05 was considered statistically significant.

## Results

### Circ_0001859 was highly expressed in NSCLC tissues and cells

**Figure 1:**
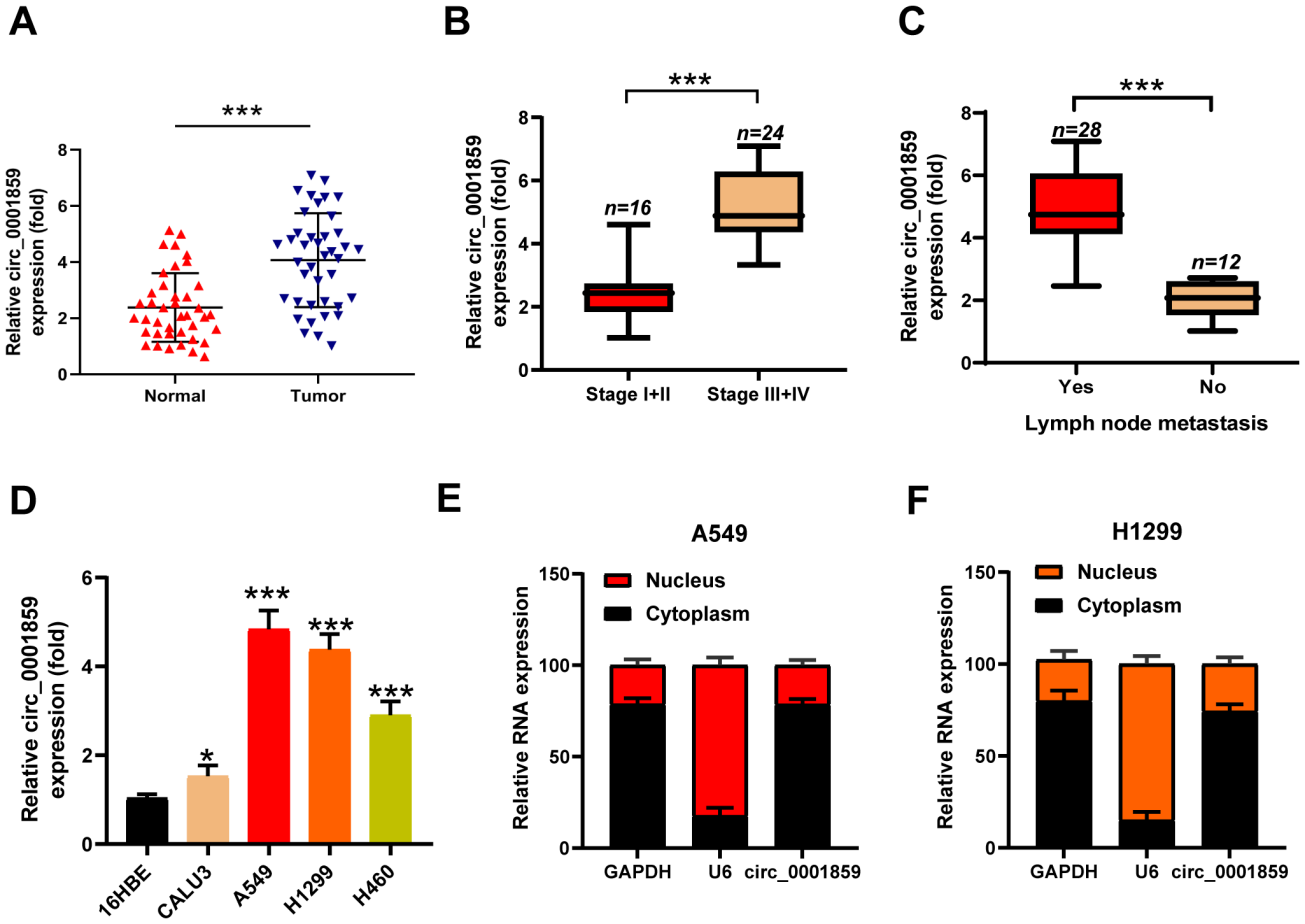
Hsa_circ_0001859 was high expressed in NSCLC tissues and cell lines. **(A)** Circ_0001859 expression levels in 40 pairs of NSCLC tissues were measured by qRT-PCR (n = 40, ***P < 0.001 versus Normal group, Student’s t-tests). **(B)** Circ_0001859 expression levels in NSCLC with different tumor stages were measured by qRT-PCR. ***P < 0.001 versus Stage I+II group. **(C)** Circ_0001859 expression levels were detected in NSCLC with lymph node metastasis (LNM) evaluated by qRT-PCR. ***P < 0.001 versus LNM group. **(D)** Circ_0001859 expression in NSCLC cell lines (CALU3, A549, H1229 and H460) were elevated compared with a normal human bronchial epithelial cell line (16HBE) measured by qRT-PCR. All experiments were performed in biological triplicates. *P<0.05, ***P < 0.001 versus 16HBE. **(E, F)** Expression levels of GAPDH, U6, and circ_0001859 in the cell cytoplasmic and nuclear were determined by qRT-PCR in A549 and H1229 cells.

Circ_0001859 was located on chr9: 37086665-37121125|+ and derived from the TCONS_I2_000287_001 transcript (**Supplementary Figure S3**). To investigate circ_0001859 expression levels, we obtained tumor tissues and paired adjacent normal tissues from patients diagnosed with NSCLC. qRT-PCR analysi**s** showed that circ_0001859 was markedly upregulated in NSCLC tissues (**Figure 1A**). We examined circ_0001859 expression across different clinical stages to determine its association with TNM staging. The results demonstrated elevated circ_0001859 expression in stage III-IV compared to stage I-II NSCLC tissues (**Figure 1B**). Consistently, higher circ_0001859 expression was detected in NSCLC tissues with LNM versus those without LNM (**Figure 1C**). We additionally analyzed four NSCLC cell lines (CALU3, A549, H1299, H460) and the normal human bronchial epithelial cell line (16HBE). The results indicated that circ_0001859 exhibited significantly higher expression in cancer cell lines, particularly in A549 and H1299, in comparison with 16HBE (**Figure 1D**). These variations may reflect differences in cellular origin. Due to their highest expression levels, these two cell lines were selected for subsequent functional studies. Subcellular localization analysis revealed that circ_0001859 was mainly distributed in the cytoplasm (**Figures 1E, F**). These findings suggest that circ_0001859 may serve as a biomarker for NSCLC.

### Circ_0001859 silencing restrained NSCLC cells proliferation, invasion and migration

**Figure 2:**
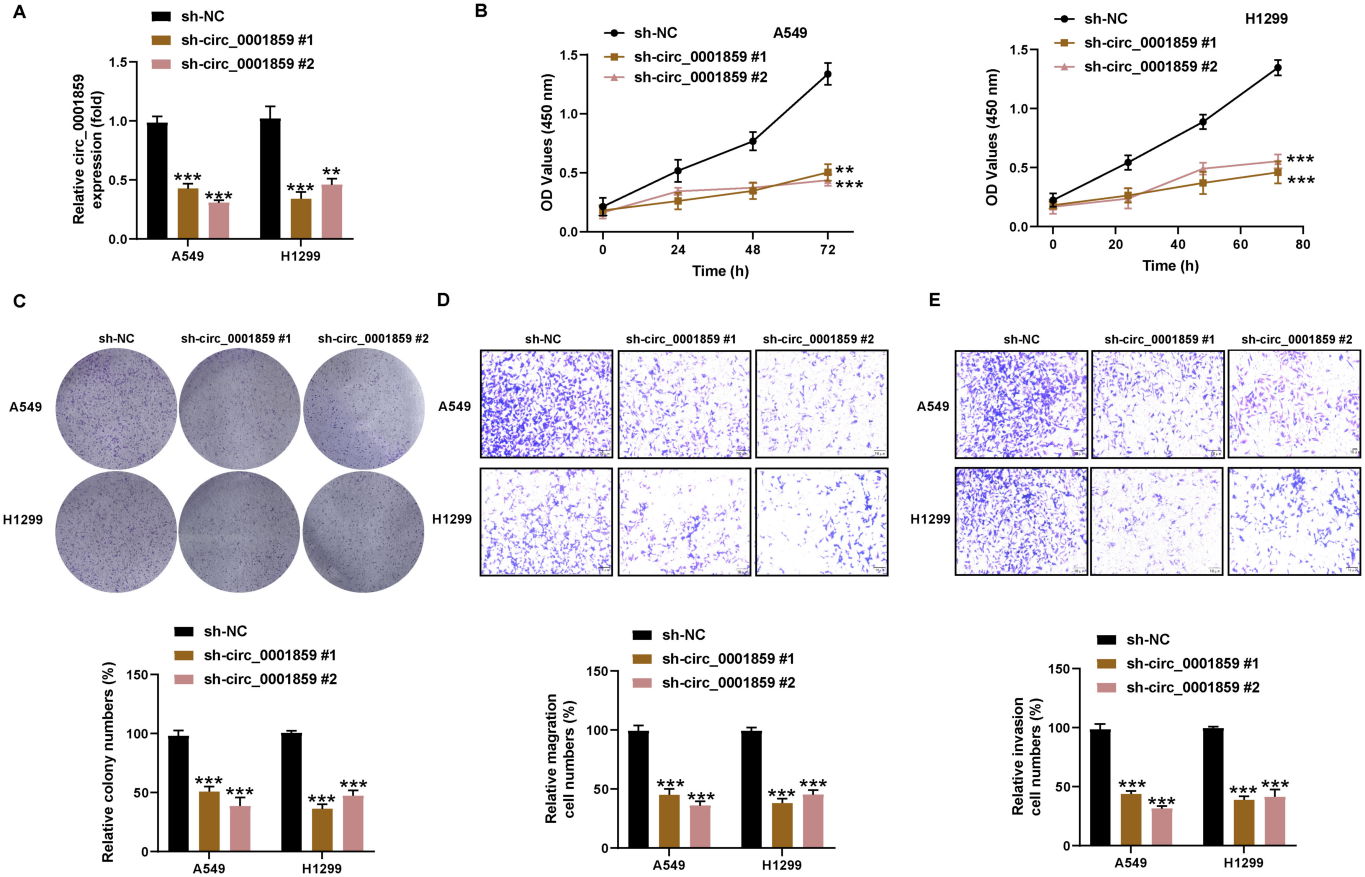
Circ_0001859 silencing inhibited proliferation, migration and invasion of NSCLC cells. **(A)** The knockdown efficiency of circ_0001859 in A549 and H1229 cells were tested by qRT-PCR. **(B, C)** Cell proliferation of A549 and H1229 cells transfected with sh-circ_0001859 (sh-NC, sh-circ_0001859#1 and sh-circ_0001859#2) determined by CCK-8 assays and colony formation assays. **(D, E)** Cell migration **(D)** and invasion **(E)** of A549 and H1229 cells transfected with sh-circ_0001859 (sh-NC, sh-circ_0001859#1 and sh-circ_0001859#2) were investigated with transwell assay. All experiments were performed in biological triplicates. **P<0.001, ***P < 0.001 versus sh-NC group.

To ascertain whether circ_0001859 contributed to NSCLC malignant progression, we successfully knocked down circ_0001859 expression by transfecting A549 and H1299 cells with sh-circ_0001859#1 or sh-circ_0001859#2, respectively, as illustrated in **Figure 2A**. We subsequently investigated the impact of circ_0001859 silencing on the NSCLC biological behavior. CCK-8 and colony formation assays demonstrated significantly reduced proliferation rates (**Figure 2B**) and fewer colonies (**Figure 2C**) in sh-circ_0001859 groups compared with sh-NC group. Correspondingly, showed significant inhibition of migration and invasion after circ_0001859 knockdown (**Figures 2D, E**). The findings revealed that silencing circ_0001859 inhibited the proliferation of NSCLC cells and altered their metastatic behavior in terms of migration and invasion *in vitro*.

### Circ_0001859 acted as a molecular sponge for miR-101-3p

Using the StarBase, GSE24709, and GSE135918 databases to identify potential target miRNAs for circ_0001859, we identified three overlapping miRNAs: miR-20a-5p, miR-101-3p, and miR-20b-5p (**Supplementary Figure S1A**). Subsequently, we validated the expression levels of these miRNAs in NSCLC by silencing circ_0001859. Our analysis revealed a significant upregulation of miR-101-3p in both A549 and H1299 cell lines, with miR-20a-5p displaying only a minor difference in A549 cells and miR-20b-5p showing no notable changes (**Supplementary Figures S1B–D**). Therefore, miR-101-3p was selected for further experimental investigations.

**Figure 3:**
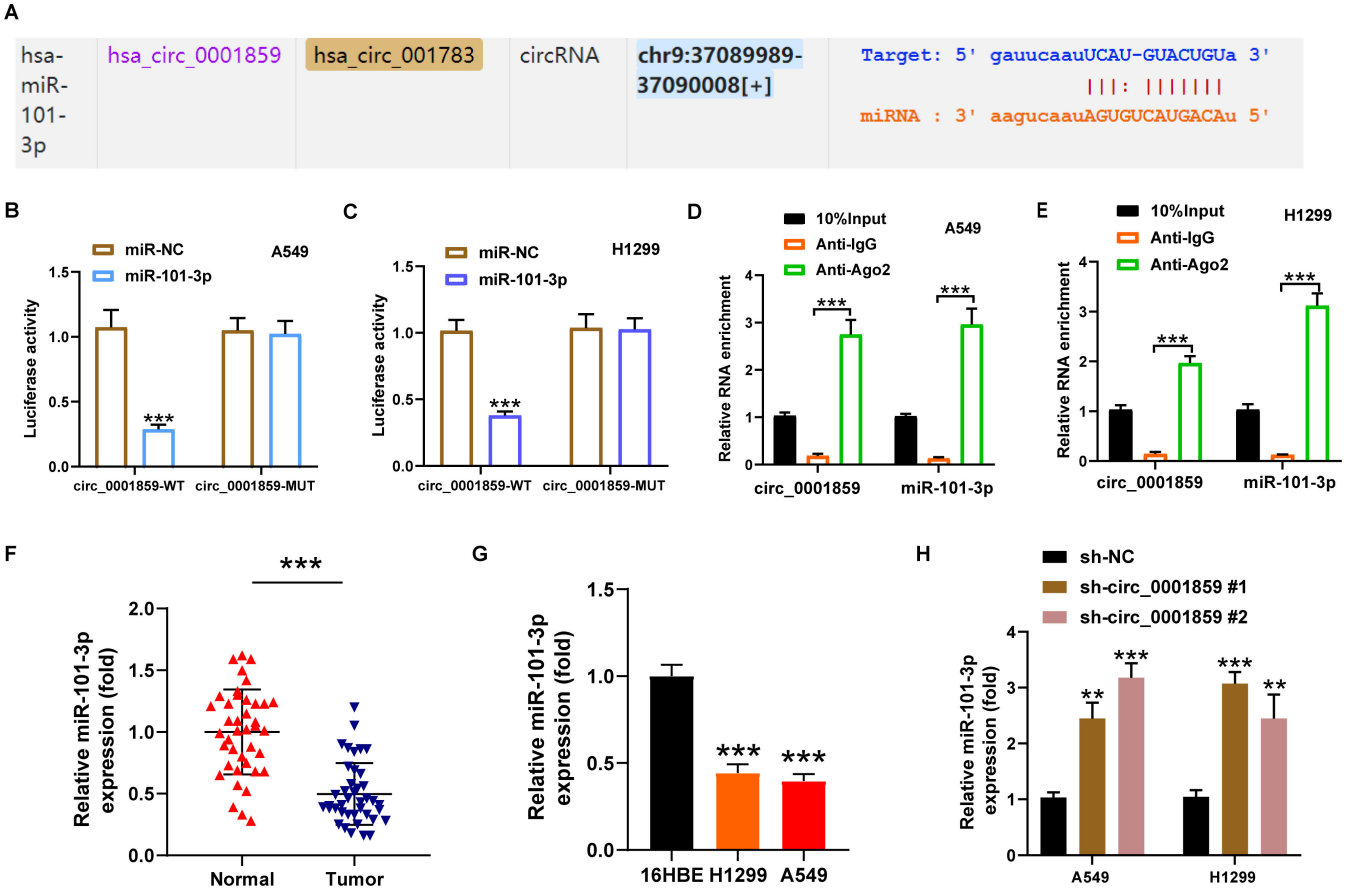
Circ_0001859 directly regulated miR-101-3p. **(A)** The complementary binding sites of miR-101-3p and circ_0001859 were predicted by online bioinformatics algorithm StarBase. **(B, C)** The relationship between circ_0001859 and miR-101-3p examined by luciferase reporter assay in A549 and H1229 cells. ***P < 0.001. versus miR-NC group. **(D, E)** The relationship between circ_0001859 and miR-101-3p confirmed by RNA immunoprecipitation (RIP) assay in A549 and H1229 cells. ***P < 0.001. versus Anti-lgG group. **(F, G)** MiR-101-3p expression in NSCLC tissues **(F)** and cells **(G)** were measured by qRT-PCR. ***P < 0.001 versus Normal group, ***P < 0.001 versus 16HBE. **(H)** MiR-101-3p expression level in A549 and H1229 cells after circ_0001859 knockdown. All experiments were performed in biological triplicates. All experiments were performed in biological triplicates. **P < 0.01, ***P < 0.001 versus sh-NC group.

Bioinformatic analysis revealed the presence of 10 miR-101-3p binding sites within circ_0001859 sequence, indicating that miR-101-3p was a potential target of circ_0001859 (**Figure 3A**). The prediction was further validated through dual-luciferase reporter and RIP experiments. Compared with the miR-NC groups, miR-101-3p overexpression suppressed the circ_0001859-Wt luciferase activities. In contrast, no significant difference in luciferase activity was observed for circ_0001859-MUT (**Figures 3B, C**). Previous studies have demonstrated that AGO2 mediated the binding between circular RNA and miRNA. Consequently, RIP analysis was performed, revealing circ_0001859 and miR-101-3p were markedly upregulated within the AGO2 immunoprecipitation complex (**Figures 3D, E**). Consistent with these findings, miR-101-3p was downregulated in NSCLC tissues and cell lines (**Figures 3F, G**). Significantly, correlation analysis revealed a strong inverse relationship between circ_0001859 and miR-101-3p expression levels (**Supplementary Figure S4A**). qRT-PCR analysis demonstrated that silencing circ_0001859 resulted in an elevation in miR-101-3p levels in NSCLC cells (**Figure 3H**). Overall, circ_0001859 could function as a miR-101-3p sponge, exerting negative regulation on its expression in NSCLC cells.

### MMP1 was a target gene of miR-101-3p

**Figure 4:**
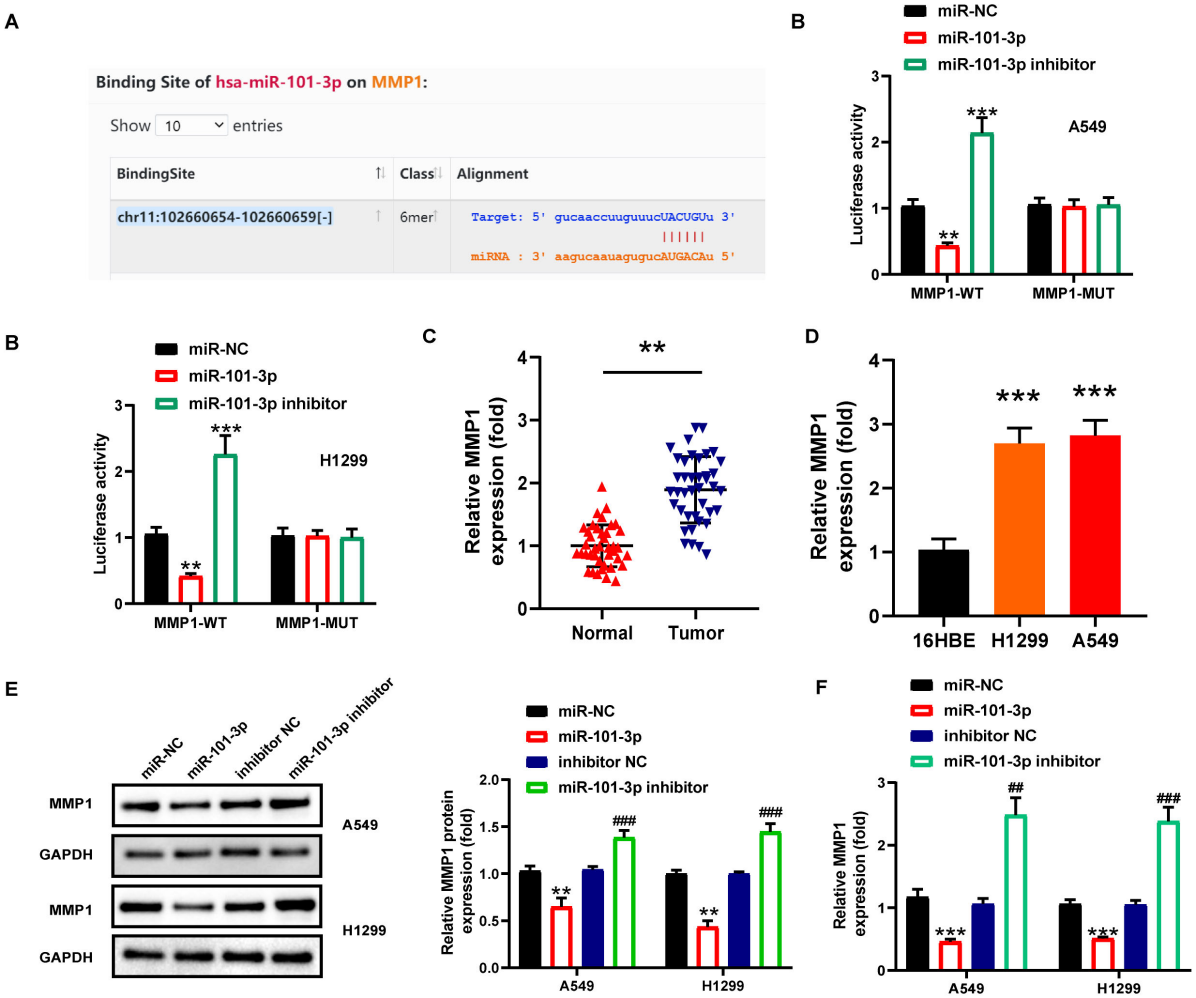
*MMP1* was a downstream target of miR-101-3p. **(A)** The complementary sequence of miR-101-3p and 3’−untranslated regions of *MMP1* predicted by the StarBase. **(B)** The relationship between *MMP1* and miR-101-3p was confirmed by luciferase reporter assay in A549 and H1229 cells. C-D *MMP1* expression in NSCLC tissues **(C)** and cells **(D)** were measured by qRT-PCR. **P < 0.001 versus Normal group, ***P < 0.001 versus 16HBE. **(E)** *MMP1* protein expression level in A549 and H1229 cells after miR-NC, miR-101-3p mimic, miR-inhibitor, miR-101-3p inhibitor transfection. **(F)** *MMP1* mRNA expression level in A549 and H1229 cells after miR-NC, miR-101-3p mimic, miR-inhibitor, miR-101-3p inhibitor transfection. All experiments were performed in biological triplicates. **P<0.01, ***P<0.001 versus miR-NC group; ^##^ *P*<0.01, ^###^ *P*<0.001 versus inhibitor-NC.

The MMP gene family, widely implicated in tumor progression, has been well-characterized in NSCLC, particularly MMP2 and MMP9 which play pivotal roles in metastasis and invasion (27). We therefore focused our investigation on this gene family. Utilizing the StarBase database, we conducted an analysis on the binding sites of miR-101-3p within the MMPs gene family, identifying six distinct MMPs genes (**Supplementary Figure S2A**), and further evaluated them using the GEPIA database. Our analysis revealed that MMP1 and MMP15 displayed significant upregulation in NSCLC, whereas MMP19 exhibited downregulation. The expression levels of the remaining three genes did not show statistical significance (**Supplementary Figure S2B**). Of these, only MMP1 demonstrated prognostic significance (**Supplementary Figure S2C**), prompting its selection for further study. Bioinformatic prediction using StarBase2.0 identified MMP1 as a functional target of miR-101-3p, with six predicted binding sites between them (**Figure 4A**). Dual-luciferase reporter gene assay demonstrated that, upon transfection with miR-101-3p mimic, MMP1-WT luciferase activity exhibited a significant decrease in A549 and H1299 cells. In contrast, the miR-101-3p-inhibitor groups showed substantial increase in MMP1-WT luciferase activities, while no notable alteration was observed in MMP1-MUT luciferase activities (**Figure 4B**). Furthermore, *MMP1* was significantly upregulated in NSCLC tissues and cells (**Figures 4C, D**). To further investigate the interrelationships among *MMP1*, miR-101-3p, and circ_0001859, Spearman correlation analysis demonstrated a statistically significant inverse correlation between MMP1 and miR-101-3p expression levels, coupled with a strong positive correlation between MMP1 and circ_0001859 expression, thereby substantiating their regulatory network in NSCLC pathogenesis (**Supplementary Figures S4B, C**). Following transfection with NC mimic, miR-101-3p mimic, inhibitor NC, or miR-101-3p inhibitor into A549 and H1299 cells. **Figures 4E, F** demonstrated that protein mimic transfection reduced MMP1 protein and mRNA expression. Conversely, transfecting miR-101-3p inhibitor yielded opposite outcomes. Altogether, these results indicated that miR-101-3p targeted MMP1 and decreased its expression in NSCLC cells.

### Circ_0001859 indirectly regulated MMP1 via sponging miR-101-3p and influenced tumorigenesis in NSCLC

**Figure 5:**
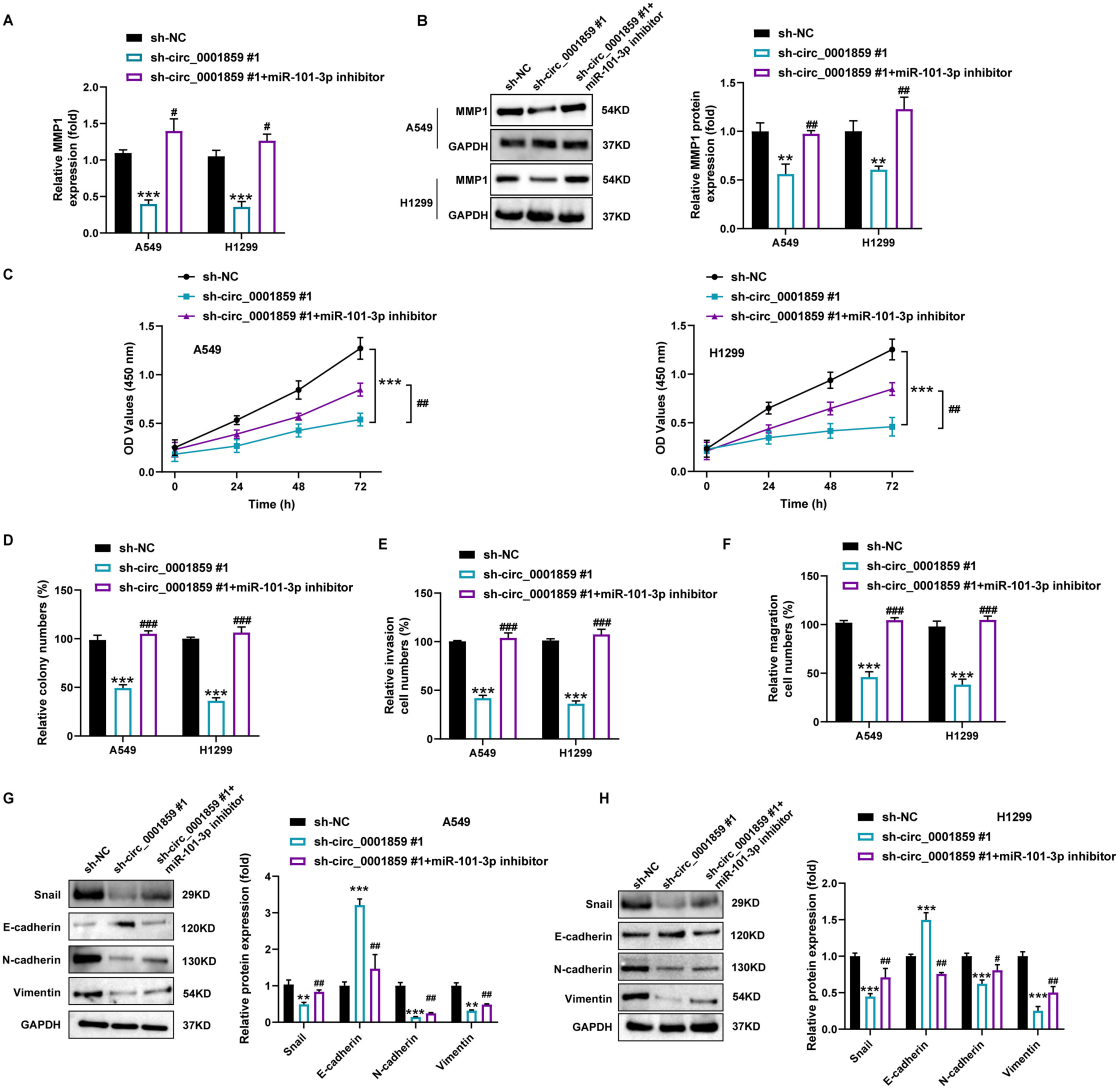
Circ_0001859 knockdown inhibited proliferation, migration and invasion of NSCLC cells via regulating miR-101-3p/*MMP1* pathway. **(A)** *MMP1* mRNA expression in A549 and H1229 cells after sh-NC, sh-circ_0001859#1, sh-circ_0001859#1+miR-101-3p inhibitor transfection. **(B)** *MMP1* protein expression in A549 and H1229 cells after sh-NC, sh-circ_0001859#1, sh-circ_0001859#1+miR-101-3p inhibitor transfection. **(C, D)** Cell viability of A549 and H1229 cells transfected with sh-NC, sh-circ_0001859#1, sh-circ_0001859#1+miR-101-3p inhibitor determined by CCK-8 assays and colony formation assays. E-F, Cell invasion **(E)** and migration **(F)** of A549 and H1229 cells transfected with sh-NC, sh-circ_0001859#1, sh-circ_0001859#1+miR-101-3p inhibitor were investigated with transwell assay. **(G, H)** Protein levels of E-cadherin, N-cadherin, Vimentin, and Snail in A549 **(G)** and H1299 **(H)** cells transfected with sh-NC, sh-circ_0001859#1, sh-circ_0001859#1+miR-101-3p inhibitor were measured by western blot. All experiments were performed in biological triplicates. **P<0.01, ***P<0.001 versus sh-NC group; ^#^ *P*<0.05, ^##^ *P*<0.01 versus sh-circ_0001859#1 group.

To determine whether circ_0001859 regulated NSCLC progression through the miR-101-3p/MMP1 axis, we performed rescue experiments. As shown in **Figures 5A, B**, circ_0001859 silencing significantly reduced MMP1 expression at both transcriptional and translational levels, an effect that was reversed by miR-101-3p inhibition. Moreover, miR-101-3p inhibitor transfection partially restored the sh-circ_0001859-induced suppression of cell proliferation (**Figure 5C**) and colony formation (**Figure 5D**). Similarly, transwell assays demonstrated that miR-101-3p inhibitor attenuated the sh-circ_0001859-mediated inhibition of cell migration and invasion (**Figures 5E, F**).

Furthermore, circ_0001859 silencing downregulated Snail, N-cadherin, and Vimentin expression while upregulating E-cadherin levels in A549 and H1299 cells (**Figures 5G, H**). Notably, EMT-related protein changes were reversed by miR-101-3p inhibition. These findings collectively demonstrated that the circ_0001859/miR-101-3p axis modulates MMP1 expression and thereby regulates the proliferative, migratory, invasive, and EMT properties of NSCLC cells.

### Circ_0001859 downregulation inhibited tumor growth *in vivo*.

**Figure 6:**
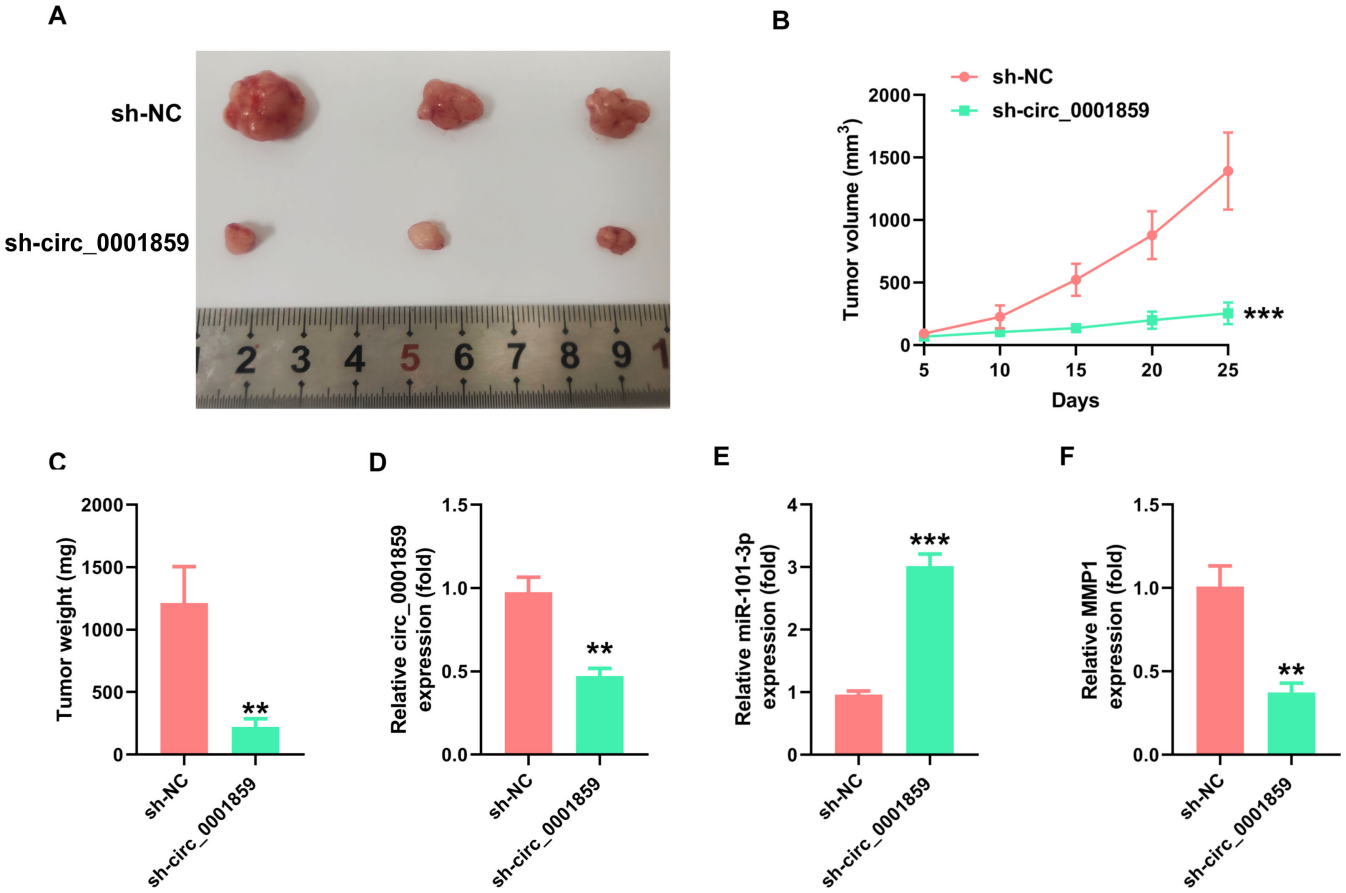
Silencing of circ_0001859 inhibited tumor growth *in vivo*. **(A, B)**. Tumor volume was detected after silencing circ_0001859. **(C)** Tumor weight was detected in each group after the mice were sacrificed. **(D-F)** The expression of circ_0001859, miR-101-3p, and MMP1 was examined in each group via qRT-PCR. All experiments were independently repeated three times, and the data are expressed as the mean ± standard deviation. **P < 0.01; ***P < 0.001 versus the sh-NC group).

By establishing a mouse xenograft model, we examined the impact of circ_0001859 on mouse xenografts. Mice were divided into two groups and injected with stably transfected A549 cells with sh-NC or sh-circ_0001859#1. Circ_0001859 silencing led to significantly reduced tumor volume and weight (**Figures 6A-C**). Consistent with this observation, circ_0001859 expression levels in tumor tissues were markedly decreased in the knockdown groups (**Figure 6D**). Importantly, decreased circ_0001859 levels correlated inversely with upregulated miR-101-3p expression (**Figure 6E**), confirming the regulatory mechanism. qRT-PCR analysis revealed substantial inhibition of *MMP1* expression in the circ_0001859 knockdown group when compared to the sh-NC groups (**Figure 6F**). Notably, our study pioneers the identification of the circ_0001859/miR-101-3p/MMP1 regulatory network as a promising diagnostic biomarker and therapeutic target for NSCLC intervention.

## Discussion

Global estimates from the World Health Organization (WHO) indicate that NSCLC constitutes 80% to 85% of all LC cases, representing a major contributor to global cancer-related mortality. Owing to the inconspicuous symptoms, limited early diagnostic techniques, and delayed diagnosis, roughly 75% patients were already in an advanced disease stage, which included distant metastasis upon diagnosis, thus missing the optimal chance for surgical intervention (28, 29). Hence, investigating the molecular mechanisms associated with the NSCLC development and elucidating potential therapeutic targets hold significant importance. Accumulating evidence has demonstrated the functional involvement of circRNAs in NSCLC progression through diverse regulatory networks (30). One notable investigation focused on circHERC1, which regulated the miR-142-3p/HMGB1 axis and activated the MAPK/ERK and NF-κB pathways, thereby facilitating NSCLC development (31). CircSATB2 was upregulated in NSCLC cells and tissues and directly binded to miR-326 to regulate FSCN1 expression, thereby influencing disease progression (32). In this research, we observed circ_0001859 was significantly upregulated in both NSCLC tissues and cell lines. Moreover, we analyzed the correlation between circ_0001859 and patients clinical characteristics. Furthermore, its expression levels correlated with clinical characteristics, showing significant overexpression in tumors of intermediate-to-advanced stages and those with LNM. To date, the role of circ_0001859 in NSCLC has yet to be fully investigated. Consequently, we effectively suppressed circ_0001859 expression via cell transfection techniques and substantiated its functional impact through rigorous experimental validation. Notably, silencing circ_0001859 impeded the malignant progression of NSCLC, highlighting its potential as a diagnostic biomarker for this disease.

The majority of circRNAs that regulate tumor progression function as miRNA sponges to indirectly control downstream target mRNA (33, 34).Numerous studies have confirmed the crucial role played by the circRNA-miRNA-mRNA axis in regulating the NSCLC malignant progression (35, 36). Using the StarBase 2.0 database, we predicted and subsequently validated that circ_0001859 targets miR-101-3p, with their negative regulatory relationship confirmed by luciferase reporter and RIP assays. Previous reports have established miR-101-3p as a tumor-suppressive miRNA (37, 38). Previous reports have established miR-101-3p as a tumor-suppressive miRNA (39), Cholangiocarcinoma (40), and oral cancer (41). For instance, miR-101-3p expression was significantly higher in plasma exosomes from medulloblastoma patients than in healthy groups, and played a vital role in tumorigenesis by targeting the FOXP4 gene (42). Liang et al. demonstrated that miR-101-3p competitively bind to LncRNA PTAR, regulating the expression of ZEB1, which induced tumorigenicity and EMT in ovarian cancer cells (43). Ding et al. demonstrated that miR-101-3p, a tumor suppressor gene, which was regulated by circ-MEMO1 that influenced aerobic glycolysis and impacted tumor progression (44). This study was the pioneering investigation between circ_0001859 and miR-101-3p, thereby providing a theoretical basis for the NSCLC carcinogenic mechanism.

Similarly, we demonstrated that MMP1 was a downstream target of miR-101-3p. MMP1 has been reported to exhibit oncogenic roles in various malignant tumors, including Uveal melanoma (45), Liver cancer (46), Cervical squamous cell carcinoma (47) and Colorectal Cancer (48). In the pathological processes of these cancers, MMP1 shows abnormally high expression levels regulated by upstream genes and pathways. Additionally, we confirmed that MMP1 protein expression was increased upon miR-101-3p knockdown. Therefore, we investigated the specific mechanism by which circ_0001859 regulated MMP1. Rescue experiments revealed that miR-101-3p knockdown rescued the inhibitory effect of circ_0001859 silencing on MMP1 expression in NSCLC cells, and partially restored the inhibitory effect of circ_0001859 knockdown on the NSCLC malignant progression. Furthermore, the downregulation or upregulation of N-cadherin, Snail protein, E-cadherin and vimentin were eliminated by co-transfection with miR-101-3p inhibitor. Moreover, the knockdown of circ_0001859 remarkably suppressed tumor size and growth, suppressed MMP1 expression and promoted miR-101-3p expression in nude mice. Through *in vivo* and *in vitro* studies, the precise mechanism of the circ_0001859/miR-101-3p/MMP1 regulatory network was elucidated.

Nonetheless, it is imperative to acknowledge various limitations present in this study. The collected data primarily derived from research involving both humans and animals, with validation limited to a small number of tumor samples and mice. Such constraints could adversely affect the relevance and precision of this findings. Therefore, these limitations must be considered before extrapolating the results to clinical practice, and further research with larger clinical cohorts and additional animal models is needed. Despite these limitations, the significance of our findings should not be overlooked. This study provides the first evidence for the involvement of the circ_0001859/miR-101-3p/MMP1 axis in NSCLC. However, additional studies are necessary to fully evaluate the potential of circ_0001859 as a clinical biomarker for NSCLC.

## Conclusions

Overall, our research findings demonstrated that circ_0001859 engaged in regulating NSCLC growth and metastasis via the miR-101-3p/MMP1 pathway. Circ_0001859 has the potential to serve as a biomarker and therapeutic target for NSCLC. However, further investigation is warranted to validate these results and evaluate the clinical applicability of circ_0001859.

## Data Availability

The original contributions presented in the study are included in the article/**Supplementary Material**. Further inquiries can be directed to the corresponding authors.
