# Supplementary figures and images for: Hsa_circ_0001859 promotes NSCLC progression through the miRNA-101-3p/MMP1 axis
Source: Front Oncol. 2025 Jul 1;15:1568367. doi: 10.3389/fonc.2025.1568367 (PMC12259449; doi:10.3389/fonc.2025.1568367)

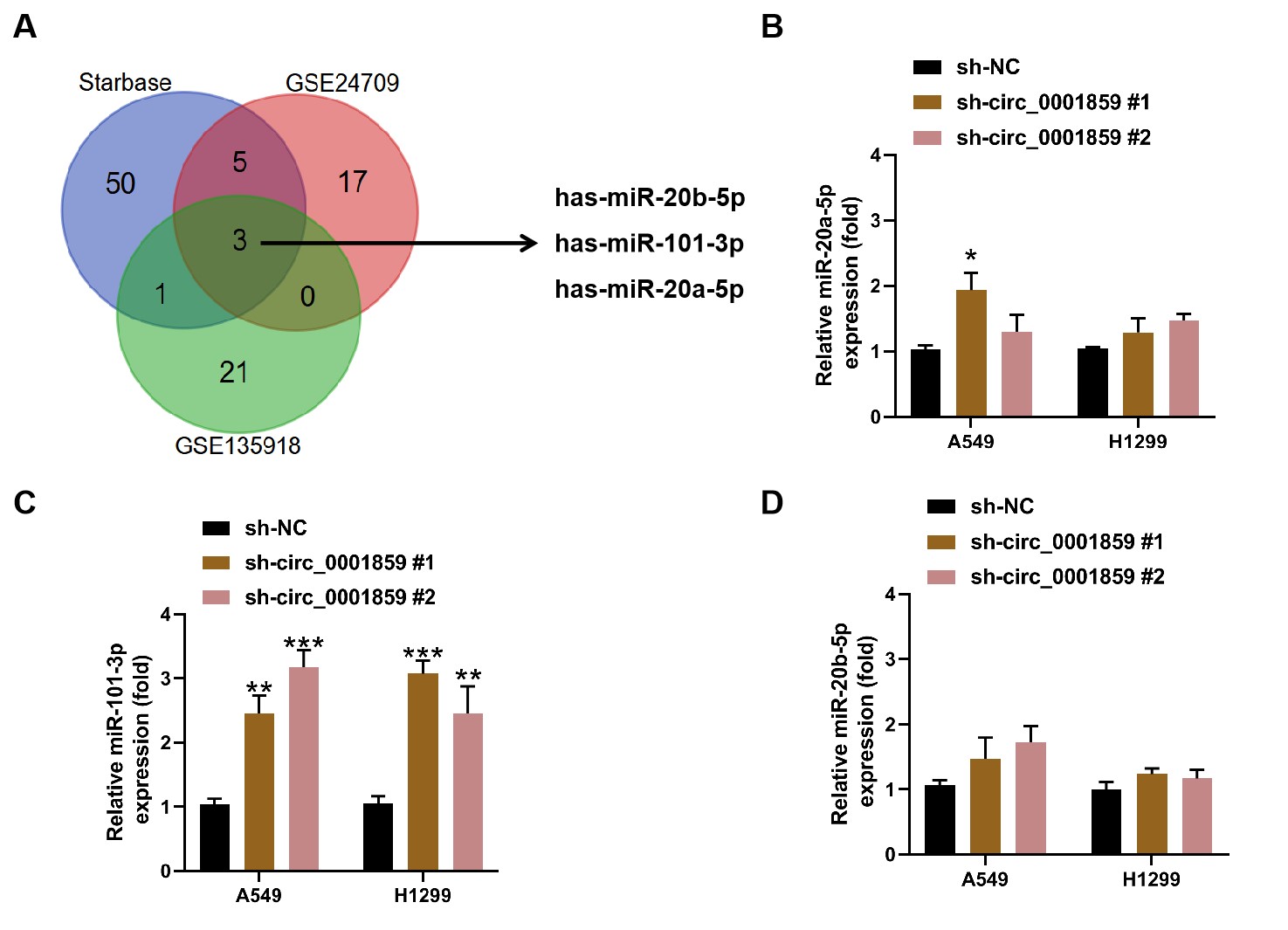

Supplement: Supplementary file 1 [file Image1.jpg]

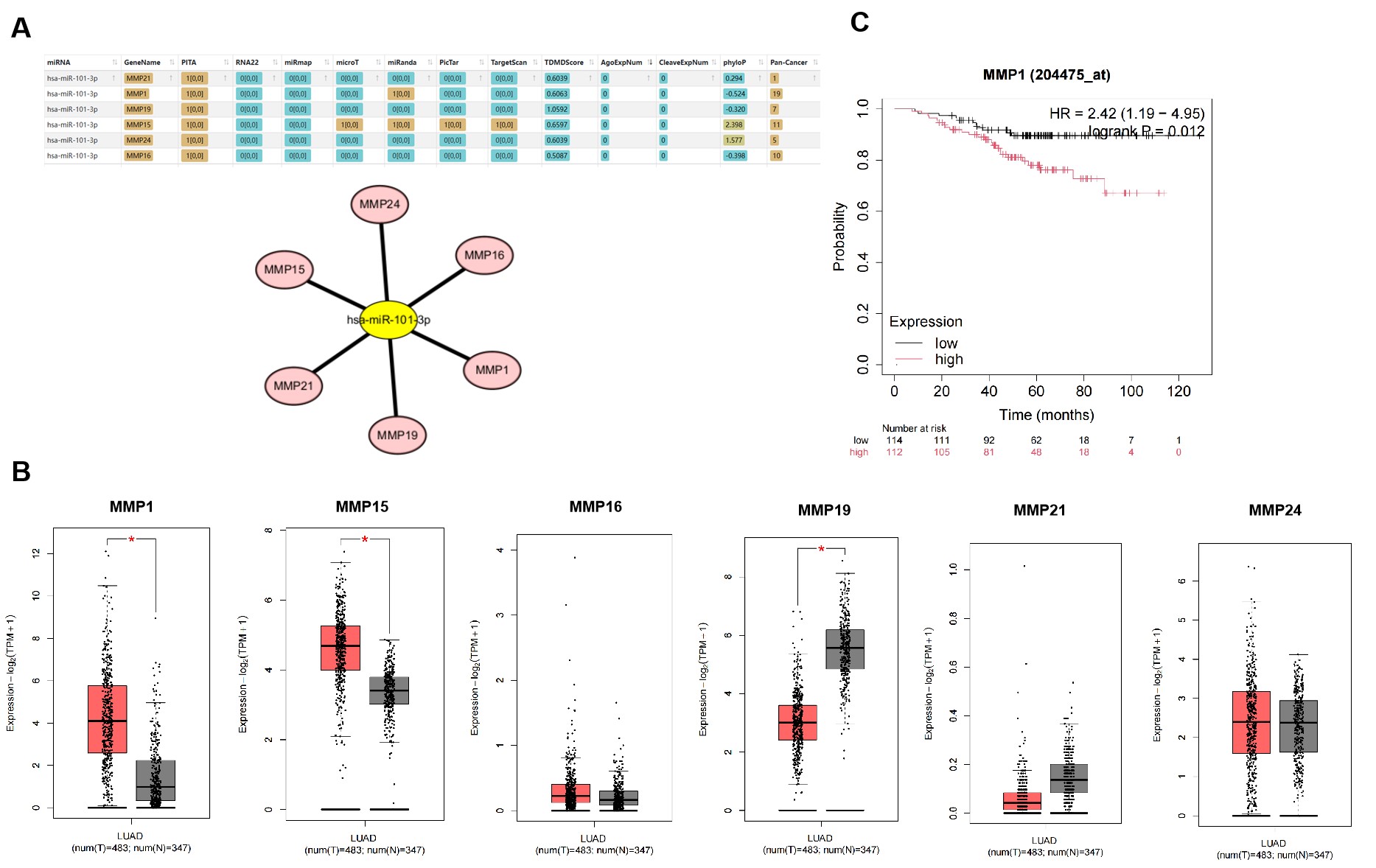

Supplement: Supplementary file 2 [file Image2.jpg]

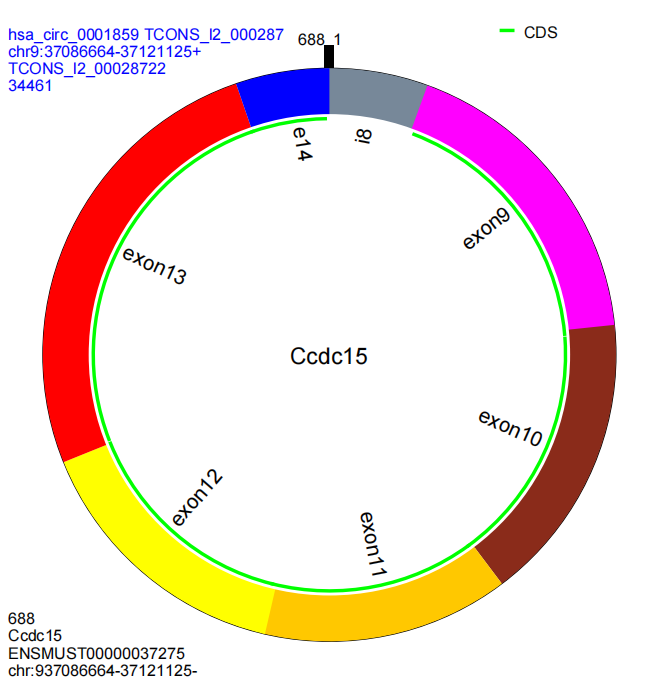

Supplement: Supplementary file 3 [file Image3.png]

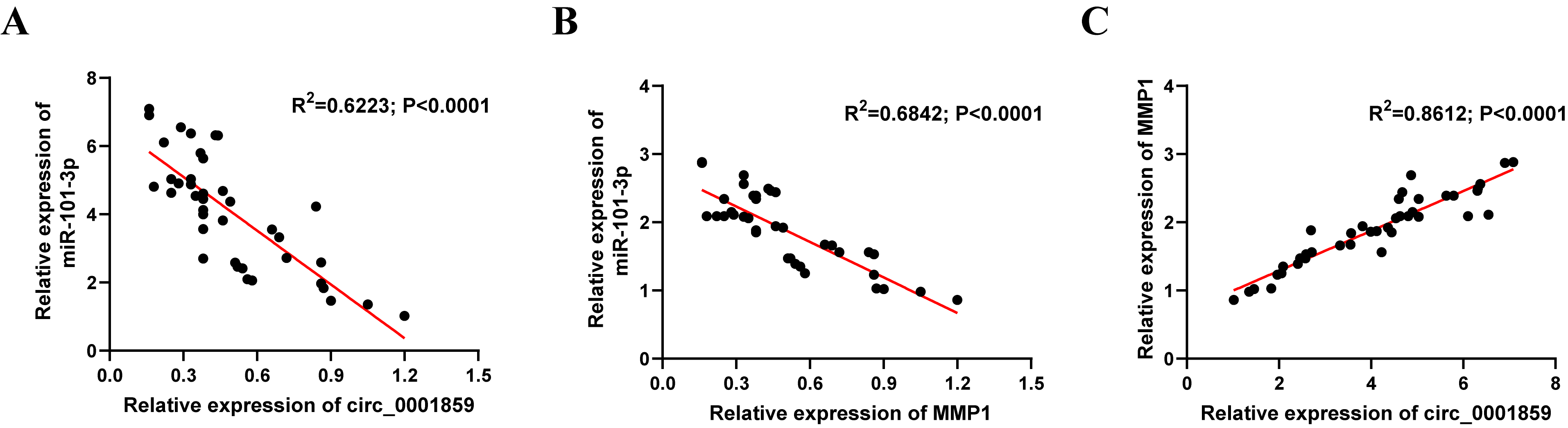

Supplement: Supplementary file 4 [file Image4.tif]
