# Supplementary material for: Hsa_circ_0001859 promotes NSCLC progression through the miRNA-101-3p/MMP1 axis
Source: Front Oncol. 2025 Jul 1;15:1568367. doi: 10.3389/fonc.2025.1568367 (PMC12259449; doi:10.3389/fonc.2025.1568367)
Supplement: Supplementary file 5 [file Table1.docx]

Table S1. Primer sequences used for cell transfection and luciferase constructs

| Name | Sequence |
| --- | --- |
| sh-circ_0001859#1 | 5′-TGGACTATCTGGGCATAGGAA -3′ |
| sh-circ_0001859#2 | 5′- AGACTTGGACTATCTGGGCAT-3′ |
| sh-NC | 5′-GCTGATCTGGGCATACCTTC-3′ |
| miR-101-3p mimics | 5′- UACAGUACUGUGAUAACUGA-3′ |
| miR-101-3p inhibitor | 5′-UCAGUUAUCACAGUACUGUA-3′ |
| NC mimics | 5ʹ-CAGUACUUUUGUGUAGUACAA-3ʹ |
| NC inhibitor | 5ʹ-UUCUCCGAACGUGUCACGUTT-3ʹ |
| circ_0001859-WT | 5ʹ- GAUUCAAUUCAUGUACUGUA-3ʹ |
| circ_0001859-MUT | 5ʹ- GAUUCAAUUCAUCAUGACUA-3 |
| MMP1-WT | 5ʹ-GUCAACCUUGUUUCUACUGUU-3 |
| MMP1- MUT | 5ʹ- GUCAACCUUGUUUCAUGACUU -3 |
